# Moving percentage of failed delta checks: a novel patient-based quality control model

**DOI:** 10.11613/BM.2026.030701

**Published:** 2026-08-10

**Authors:** Niyazi Samet Yilmaz, Bayram Sen, Sena Turkmen, Hikmet Can Çubukçu, Ozlem Gulbahar

**Affiliations:** 1 Department of Medical Biochemistry, Gazi University Faculty of Medicine, Ankara, Turkey; 2 Department of Clinical Biochemistry, Recep Tayyip Erdogan University Training and Research Hospital, Rize, Turkey; 3 Rare Diseases Department, General Directorate of Health Services, Turkish Ministry of Health, Ankara, Turkey

**Keywords:** patient-based quality control, delta check, systematic error, quality assurance, quality control, biological variation

## Abstract

**Introduction:**

For measurands with a low index of individuality (II), delta check is crucial. If delta percentage change (DPC) values in different patients consecutively exceed the reference change value (RCV), it may indicate an analytical problem. Similarly to moving sum (MovSuM), percentage of DPCs exceeding RCV can be used as a quality control model by comparing it with a certain threshold. This study aimed to develop a novel patient-based quality control (PBQC) model using delta checks and evaluate its performance.

**Materials and methods:**

The study was performed at Gazi University Biochemistry Laboratory. The model was tested on creatinine (measured on Advia Chemistry Systems using the alkaline picrate method), with pooled-CV_a_ = 2.5%, CV_i_ = 4.7%, and RCV = 19%. If DPC > RCV, it was counted as a violation, *i.e.* failed delta check (FDC). The percentage of violations was calculated for every 20 consecutive patients with previous results over the past year. Similarly to MovSuM, when a new result is added, moving percentage of failed delta checks (MPFDC) removes the oldest data and calculates the percentage of violations continuously. Moving percentage of failed delta checks thresholds determined as 35%/30% (for positive/negative direction). After excluding dialysis patients and patients without previous results, and previous results measured with different systems, we applied truncation (truncation limits: < 27 and > 362 μmol/L). The remaining 5484 results were divided into 55 batches.

**Results:**

After applying positive and negative errors of 15%, median number of patients affected before error detection (MNPed) was 34 and 29, respectively. Median time until error detection was 18 minutes. Sensitivity and specificity were 96% and 86%, respectively.

**Conclusions:**

Moving percentage of failed delta checks, a novel RCV-based PBQC model, has high sensitivity and rapid error detection, suitable for measurands with low II.

## Graphical abstract

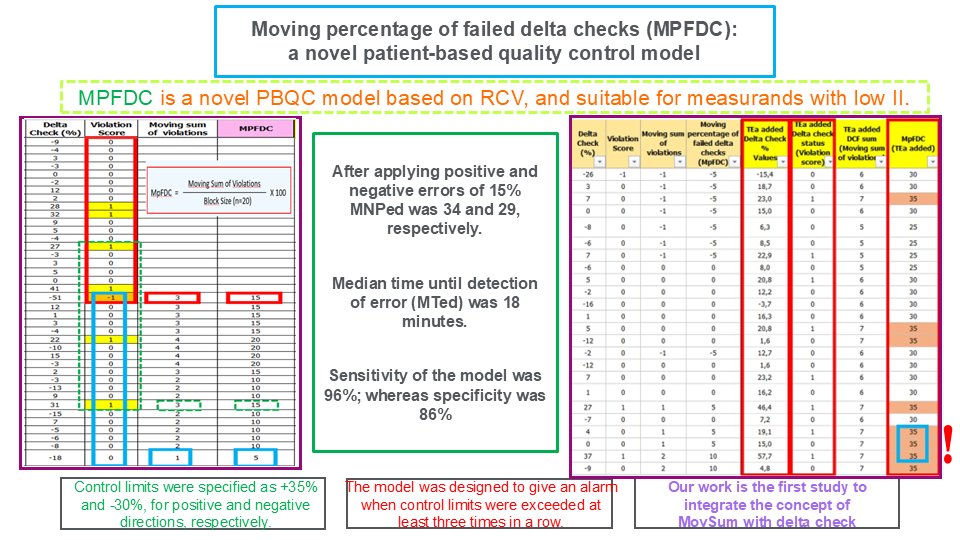

## Highlights

• Moving percentage of failed delta checks (MPFDC) is a novel patient-based quality control model based on reference change value, and suitable for measurands with low index of individuality

• MPFDC may be integrated with the laboratory information system and middleware

• MPFDC evaluates analytical performance by considering biological variation

• Appyling errors of ± 15%, median number of patients affected before error detection was 34/29 respectively and median time until error detection was 18 minutes

## Introduction

With extremely significant improvements in technical and workflow processes in the laboratory, the frequency of analytical errors has decreased over the years. However, it still has not been minimized to the desired level, and it still occurs (*1*, *2*). To cope with analytical errors, traditional quality control (QC) strategies are applied in almost all laboratories today. During traditional QC intervals, laboratories release hundreds of results; therefore, QC strategies are needed to monitor analytical quality continuously. Regarding this issue, patient-based quality control (PBQC) may be a solution. The use of PBQC is particularly important for systematic error detection and patient safety, especially for laboratories that release patient results *via* autoverification.

Recently, we have witnessed a paradigm shift and an increase in the implementation of PBQC methods (*3*, *4*). Patient-based quality control has several advantages over traditional QC including cost, commutability, and the ability to perform near real-time monitoring (*5*). Patient-based quality control approaches such as moving average (MA), moving median (MM), average of normal, moving sum (MovSum) and delta check implementations are helpful in detecting errors, whether systematic or not (*6*-*9*). However, each PBQC technique has several advantages and drawbacks. For example, MA may not perform efficiently when the data is skewed (*5*).

Delta checks, which apply for different purposes in laboratories, can also be integrated into PBQC methods (*9*). Harnessing delta check, simple and rational methods can be designed to detect systematic errors. Recently, such approaches have been developed through delta checks to detect analytical errors in the laboratory. Concepts such as average of delta (AoD), average of patient deltas, and the delta plus-minus even distribution check have been mentioned recently (*10*-*12*).

During verification of test results, one of the data that guides a clinical biochemist most is the previous results of the patients, indeed. Especially in tests with a low index of individuality (II) such as creatinine, the previous results of the patients are more important than population-based reference intervals (popRIs). The II for many measurands is lower than 0.6. When a measurand’s II is low, it is useful to follow up patients with the personalized reference intervals (prRI) rather than popRIs (*13*). For the measurands suitable for prRI, delta check implementation will be important in determining whether the person’s steady state has changed or not. Moreover, if the delta change value of a measurand consecutively exceeds the reference change value (RCV) in different patients, it may indicate an analytical problem (*14*). At this point, we can state that our study begins where delta check (*i.e.* biological variation) meets MovSum. In our model named moving percentage of failed delta checks (MPFDC), first as in the rationale of the MovSum method, binarization is applied to delta check failures (violations) in both directions. Then, the percentages of failures (delta change exceeding the RCV) in an estimated block size is used as a quality control model by constantly monitoring and comparing it with a certain threshold/cut-off. However, it needs a dynamic approach such as MovSum or MA, for monitoring the analytical quality in a timely manner.

Delta change values in some tests (such as creatinine, troponin, sodium) are important in the diagnosis and clinical course of the disease. In the College of American Pathologists (CAP) Q-Probes Study, 78% of laboratories reported using delta check for creatinine (*15*). In this study, it was seen that creatinine ranked second after sodium in terms of delta check usage frequency (*15*). To date, Risk, Injury, Failure, Loss of Kidney Function, and End-Stage Kidney Disease (RIFLE), Acute Kidney Injury Network (AKIN) and Kidney Disease: Improving Global Outcomes (KDIGO) classifications have been defined for acute kidney injury. In the definitions made by RIFLE, AKIN and KDIGO delta change value of creatinine is emphasized (*16*). In this context, with a low II value, creatinine is a very suitable test for our model. Therefore, the model was tested on creatinine.

In this study, we aimed to develop a PBQC model that enables the detection of analytical or preanalytical systematic errors using delta check and to demonstrate its performance criteria on real patient data.

## Materials and methods

The study was conducted at Gazi University Health Research and Application Hospital (GUHRAH), Core Biochemistry Laboratory, between September 2023 and November 2023. The Gazi University Health Research and Application Hospital is a tertiary care academic medical centre, which has approximately 1000 beds. Approximately 1400 creatinine test results are released in the GUHRAH Core Laboratory in a day. For the study, ethical approval was obtained from the Gazi University Faculty of Medicine Clinical Research Ethics Committee (Date: 21.03.2025, Number: 031).

The creatinine was analysed using the alkaline picrate method using the ADVIA Chemistry XPT System (Siemens Healthineers, Erlangen, Germany). For creatinine, the pooled coefficient of analytical variation (CV_a_) of four autoanalysers generating creatinine results was 2.5%. All the four analysers were calibrated just before the start of the study. Analyser comparability was not evaluated, however, median values for each analyser during the study period and strafied by day is provided at Supplementary data (Supplementary Table 1 and 2).

## Choosing the appropriate within-subject biological variation value and reference change value calculation

To determine the within-subject biological variation (CV_i_) value, all publications on creatinine were evaluated in the European Federation of Clinical Chemistry and Laboratory Medicine (EFLM) biological variation database (*17*). The publications from the last 20 years, which included serum as the sample type, mixed gender, and non-enzymatic methods were particularly examined. Since the analyser, method, gender and sample type of our laboratory are the same, CV_i_ was chosen from the EuBIVAS study as 4.7% (*18*).

The formula originally proposed by Harris and Yasaka was used to calculate RCV: RCV = 2^1/2^ x Z x (CV_A_^2^ + CV_I_^2^) ^½^ (*19*). Delta check limits were established using bidirectional Z-score (2.58 for 99% probability to achieve a ≤ 1% false rejection rate). The CV_a_ value in the formula was calculated using the results of the internal QC, with a pooled standard deviation approach. Finally, the RCV value was calculated as RCV = 19% with CV_a_ = 2.5% and CV_i_ = 4.7%.

## Editing the raw data for the model

**Figure 1:**
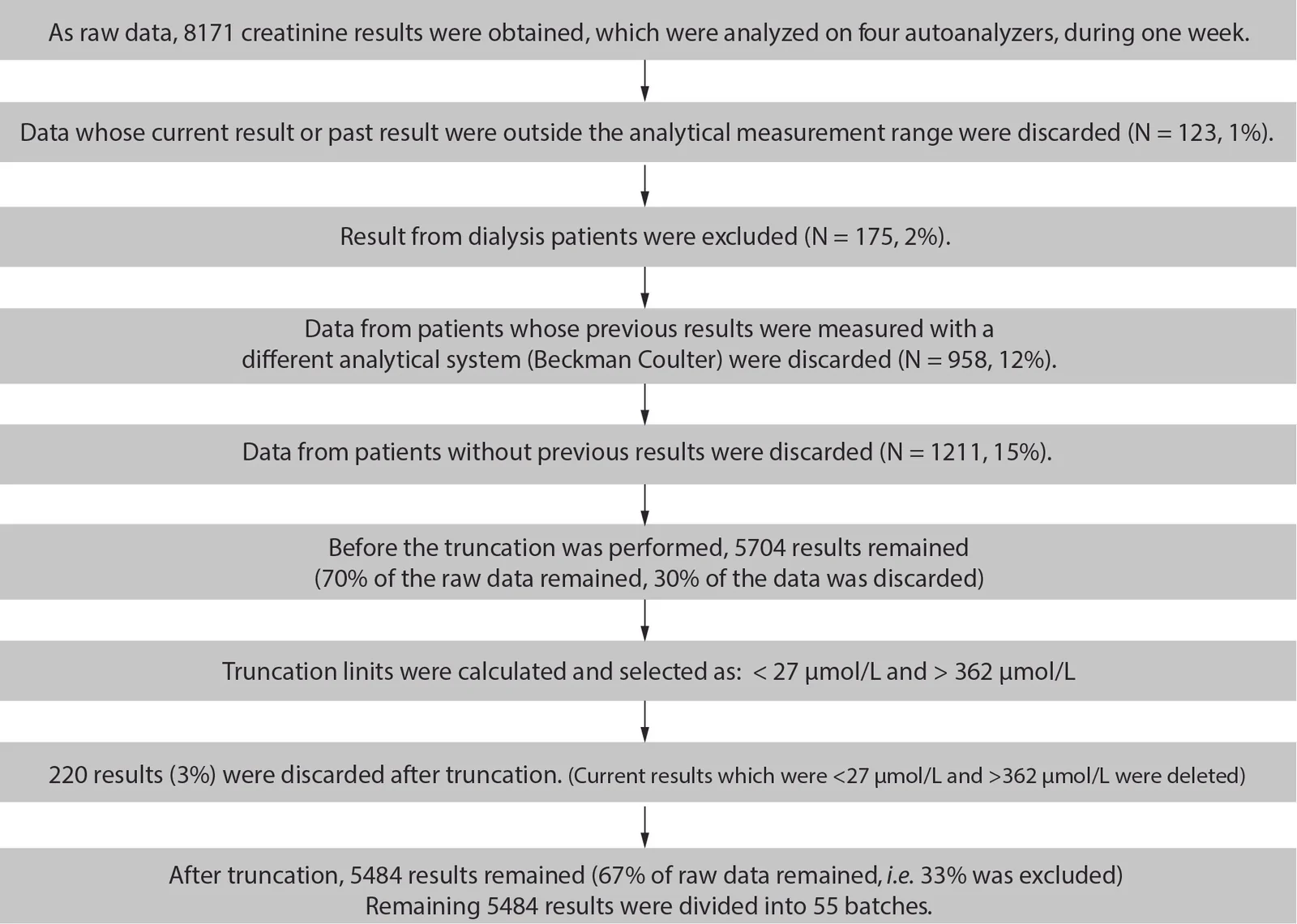
The flowchart of editing the raw data.

For PBQC models, some data should be excluded to yield better performance from the model. Thus, we performed several data processing steps on the raw data. The truncation limits were defined by excluding results below the 2nd percentile and above the 98th percentile of the data. Truncation limits were set < 27 μmol/L and > 362 μmol/L (< 0.31 mg/dL and > 4.1 mg/dL). The flowchart regarding editing the raw data is given in Figure 1.

Exclusion criteria were: 1) results from dialysis clinic; 2) current or past results outside the analytical measurement range; 3) data from patients without previous results; 4) data from patients whose previous results were measured with a different analytical system and 5) results outside of truncation limits.

Except for the exclusion criteria, all results from all autoanalysers over a one-week period were considered as inclusion criteria. No exclusions were made based on age. In the model applied for creatinine, for delta percentage change (DPC) calculation we included the patients’ previous results of up to one year, because recent studies shown CV_i_ for creatinine does not change significantly up to one year (*20*, *21*).

## The model: moving percentage of failed delta checks

The distribution of patient results for creatinine was evaluated (whether the patient results’ distribution was symmetrical or skewed) before applying the model. It was seen a skewed distribution of patient results.

Our model is inspired by an original study from Jones (*10*). Also, MPFDC is inspired by the MovSum and moving rate of positive patient results methods for the binarization process after checking whether the delta check value was greater than RCV or not (*6*, *22*).

**Figure 2:**
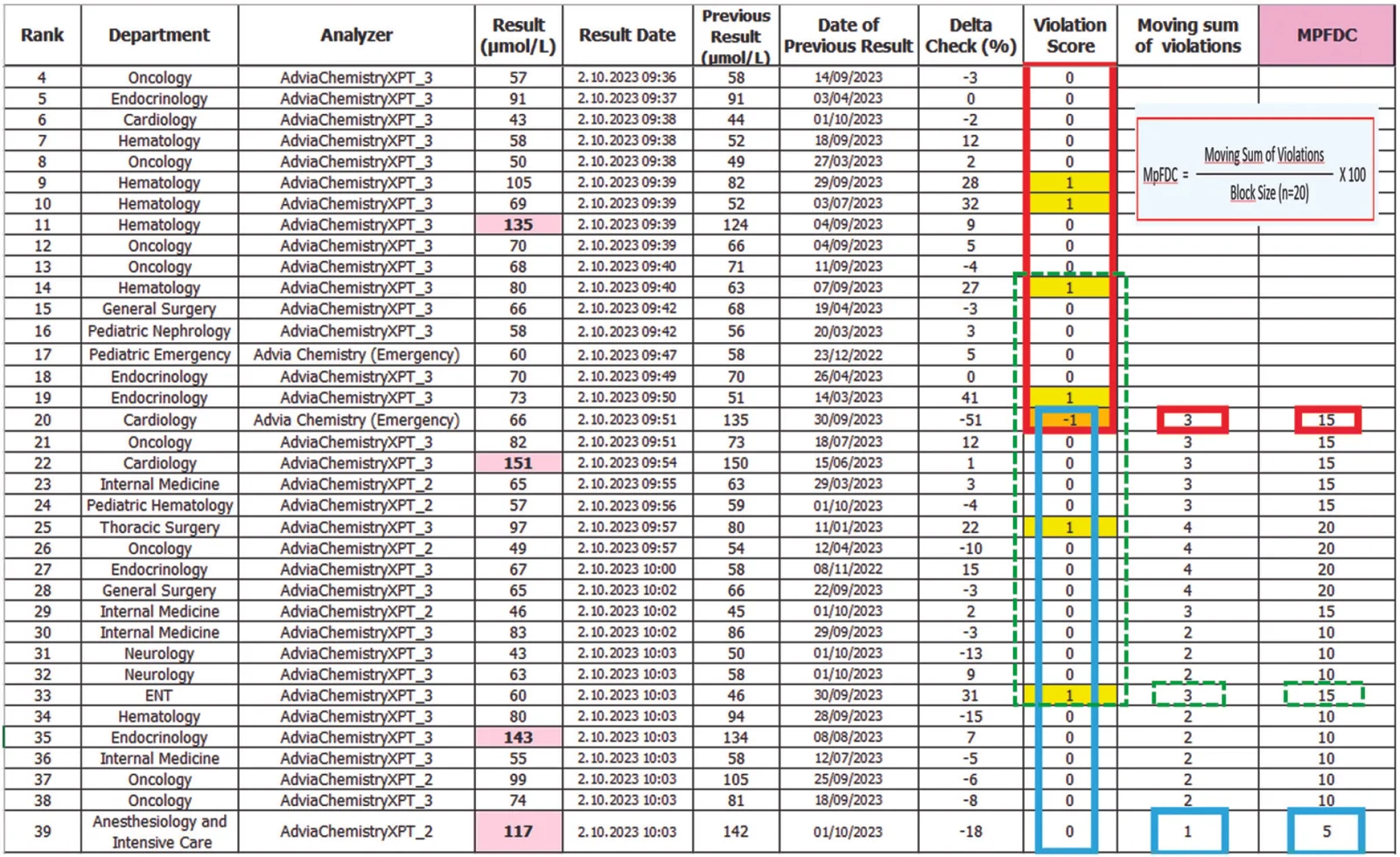
Moving percentage of failed delta checks (MPFDC) Excel sheet. Current results above the reference range are highlighted in red. The red, green dashed, and blue boxes represent different blocks. The color variation is used to distinguish between the blocks. AdviaChemistryXPT_1 (autoanalyser 1) is not presented in the figure, because this autoanalyser started to perform at 10:10 in that day.

In our model, DPC values were used to determine the change between patients’ current and previous results. If DPC > RCV for a measurand (*i.e.* creatinine), this was counted as a violation; *i.e.* failed delta check (FDC). If the violation was in the increasing direction, it was counted as + 1 point; however, if there was a decreasing direction, it was counted as - 1 point. In cases where |DPC| < |RCV| (the absolute value of DPC < RCV), it was counted as 0 point. The model was implemented with a block size of 20 consecutive patients with previous result over the past year. For each block, the arithmetic sum of violation scores (DPC > RCV) was calculated and converted into a percentage. When a new result was added, the oldest data were removed, and the moving sum of violations (moving sum of failed delta checks) was recalculated and converted into percentage accordingly. We named our method moving percentage of failed delta checks (MPFDC). The Excel implementation of the model is shown in Figure 2 and Supplementary Figure 1.

Continuously, MPFDC calculates the percentage of the number of violations in the system. Our model is based on within-subject biological variation and can reveal systematic errors.

## Establishing the model’s control limits

After designing the model, we wanted to explore the range in which our MPFDC values oscillate during a time period when analytical quality is acceptable. To prove no analytical error was present while establishing the MPFDC control limits, QC material was run for creatinine every two hours on all four autoanalysers for one week. In this way, it was proved that all the analysers were in acceptable analytical condition. Under both day-shift and night-shift conditions, QC was performed every two hours, and creatinine data for that week were evaluated. With this data generated from four autoanalysers, for the week to which the strict quality control protocol applied, the maximum MPFDC values naturally observed in the data were analysed. Afterwards, we calculated the MPFDC control limits (MPFDC thresholds/cut-offs) from this data. After evaluating the data, the control limits were specified as +35% and -30% of every 20 consecutive patients with previous result over the past year, for positive and negative directions, respectively. The data retrieved from GUHRAH’s laboratory information system were analysed via Microsoft Excel.

## Error simulation and moving percentage of failed delta checks’s performance of error detection

After establishing control limits, the model’s performance was evaluated using erroneous results. The performance of MPFDC was assessed by applying an error of 15% in both directions (positive and negative directions) to real patient data. To demonstrate how the model works, a visual of how the model detects error after applying a 15% error in the positive direction (for a random batch) was presented (Supplementary figure 1). Error has been added to the creatinine results, in accordance with the analytical performance specifications of Clinical laboratory improvement amendments (CLIA) in effect at the time the study was conducted (*23*).

All results (5484 patient results) were divided into 55 batches. Then, we introduced total allowable error (TEa; 15% error) in both positive and negative directions to every batch. Afterwards, our model’s sensitivity and specificity were checked. According to the simulations, the most appropriate control limits for the MPFDC model with the highest error detection sensitivity and specificity were + 35% and - 30% in the positive and negative direction, respectively. An important point to note here is that the system was designed to give a warning (alarm) when the model exceeded the MPFDC control limits (specified as + 35% and - 30%) at least three times in a row. In this way, the possibility of false alarms was reduced, and the specificity of the system was increased.

Error were introduced to the patient data, and the number of patient results falling between the error introduction and MPFDC alarm (*i.e.* MPFDC exceeding the established control limit three times in a row) was calculated as average number of patients affected before error detection (ANPed) and median number of patients affected before error detection (MNPed). In addition, average time until error detection in minutes (ATed), median time until error detection in minutes (MTed), average number of deltas to error detection (ANDed) and median number of deltas to error detection (MNDed) were calculated as performance metrics. For these calculations, also for sensitivity and specificity analyses, MPFDC’s performance was analysed in 55 batches spaced at 100-sample intervals. The error was applied starting from the first patient at the beginning of each batch. After detecting the error using the MPFDC, model’s performance metrics were calculated on the raw data.

### Statistical analysis

Normality of the data was evaluated for each statistical analysis. On every occasion, after Kolmogorov-Smirnov test, it was seen none of the groups had normal distribution, so nonparametric tests were performed. Kruskal-Wallis test is used for comparing the groups, and pairwise comparisons were performed with *post-hoc* Dunn test after Bonferroni correction. Descriptive values are given as number (*N*), median and interquartile range (IQR). Significance was assumed for all values where P < 0.05.

## Results

The raw data consisted of 8171 patient results. A total of 5987 (73%) of the raw data had previous results within the last year. In the raw data, 1239 (15%) results were above the reference interval. There were 164 results with critical values, corresponding to 2% of the total results.

There were four autoanalysers in our laboratory and so in the study. For each analyser, QC results are presented as mean (SD) for Level 1 and Level 2, respectively: analyser 1: 215 (5.1) and 506 (6.2) μmol/L; analyser 2: 210 (6.3) and 498 (11.3) μmol/L; analyser 3: 216 (6.1) and 510 (11.6) μmol/L; analyser 4: 215 (6.0) and 500 (13.3) μmol/L.

Analyser 1 yielded CVs of 2.4% and 1.2% for level 1 and level 2, respectively, whereas analyser 2 showed 3.0% and 2.3%. Similarly, analyser 3 demonstrated CVs of 2.8% and 2.3%, and analyser 4 showed 2.8% and 2.7% at the respective levels. The combined analysis of all analysers resulted in an overall pooled CV of 2.5%, representing the overall within-laboratory imprecision of the analytical system.

Patient results were obtained over a period of one week (five working days and night shifts, besides two weekend days). If Analyser 4 was not malfunctioning, only Analyser 4 (emergency device) was operational during night shifts from 17:00 to 08:00 and on weekends. The number of patients analysed on each autoanalyser during this one-week period was 1915 (23%), 2210 (27%), 1787 (22%) and 2259 (28%) for analyser 1, analyser 2, analyser 3 and analyser 4, respectively. Median (IQR) values of emergency department, outpatients, and inpatients were 66 (*34*), 66 (*26*) and 75 (*28*) μmol/L, respectively. The results of inpatients were higher than both patients from emergency department and outpatients (P < 0.001). No difference was found between the emergency department and outpatients. For delta check interval it was seen the median delta check interval was 14 days. Minimum delta check interval was 0 days (*i.e*. 8 hours) and maximum delta check interval was 365 days. The 25th-75th percentile of delta interval was 2-87 days.

After applying positive and negative errors of 15% to each batch, the model’s performance metrics were calculated on the raw data. Median number of patients affected before error detection (MNPed) was 34 and 29, respectively (Table 1). Median time until detection of error (MTed) was 18 minutes. Sensitivity of the model was 96%; whereas specificity was 86% (Table 1). Besides MNPed and MTed, all the other performance metrics such as ANPed, ATed, ANDed and MNDed are presented in Table 1. In addition, Table 2 shows the MPFDC application rates and delta check violation ratios in patient data where no errors were applied.

**Table 1:** Moving percentage of failed delta checks’ performance when 15% error was applied

	**Positive error applied**	**Negative error applied**
ANPed*	37	39
MNPed*	34	29
ATed (minutes)^†^	41	63
MTed (minutes)^†^	18	18
ANDed	20	24
MNDed	16	17
Total Number of Batches	55	55
Number of False Negative Batches	4	0
Number of False Positive Batches^‡^	8	
Sensitivity (%)	96	
Specificity (%)	86	
*The performance of the MPFDC calculated for raw data. ^†^Time represented as minutes, and calculated for raw data. ^‡^In the normal patient data without any errors applied, there are eight false positive batches. Six of which were in the positive direction, and two were in the negative direction. Control limit positive cut-off 35% and control limit negative cut- off - 30% were applied. ANPed - average number of patients affected before error detection. MNPed - median number of patients affected before error detection. ATed - average time until error detection. MTed - median time until error detection. ANDed - average number of deltas to error detection. MNDed - median number of deltas to error detection.		

**Table 2:** Moving percentage of failed delta checks application rates and delta check violations by patient type

	**Outpatient**	**Inpatient**	**Total**
All patients in raw data, N(%)	5694 (70%)	2477 (30%)	8171 (100%)
MPFDC (delta check) applied patients, N(%)	3302 (58%)	2182 (88%)	5484 (67%)
DPC (delta check) violations in MPFDC model when there is no error, N(%)	479 (15%)	458 (21%)	937 (17%)
Data presented in this table are raw patient data before errors were applied to the patient results. MPFDC - moving percentage of failed delta checks. DPC - delta percentage change.			

## Discussion

Here, we assessed the ability of the MPFDC model’s performance in detecting simulated errors in the real patient data. The model’s performance is quite good to detect an error of 15% in creatinine. Our model is based on within-subject biological variation and inspired by MovSum and MA methods (*6*-*8*). A key point in our study is that the model’s performance on raw patient data (real patient data) has been determined. This allowed us to see the model’s true performance across the whole patient data, regardless of whether the patient’s previous results were available. This is a valuable aspect of our study.

Indeed, there are two similar studies to our work in the PBQC literature. One of the studies similar to our study is from Cembrowski *et al.* (*11*). In their study, they combined delta check and MA to develop an average of deltas (AoD) strategy that monitors the mean delta of consecutive, intrapatient results. However, our study differs from their study because we were not interested in AoD values. Instead, after applying binarization to delta check failures (violations) and calculating the sum of violations, we used the failed delta check ratios in every 20 patients. Our study is distinct because we combined and summed delta check failures and calculated failure ratios dynamically, similar to MovSum and moving average, rather than using AoD. To our knowledge, our work is the first study to integrate the concept of MovSum with delta check (*i.e.* integrating the MovSum concept with biological variation). In this study, from delta check failures, we first tried to obtain a moving sum value considering both positive and negative directions and then calculated the percentage of this sum value in every 20 patients in a moving way.

Another similar study to ours is from Hatanaka *et al.* (*12*). They developed a dynamic PBQC model based on the variation in the distribution of delta check values in positive and negative directions. However, their model does not employ any binarization process based on the RCV value, as in our study. They based their design on positive and negative delta check values and did not compare them with a cutoff such as RCV. To our knowledge, there is no other study that applies binarization to delta check failures, then arithmetically sums the violation counts to account for the effects of both positive and negative aspects similar to MovSum, and then dynamically applies this to every 20 patients as in MA or MovSum logic. We believe that our publication synthesizes and integrates the rationale of biological variation, MA, and MovSum.

Determining the maximum time interval for applying delta checks can be estimated by considering whether physiological biological variation of the measurand is stable or not during the selected period (*21*). In general, patients’ previous results from a maximum of three months ago are used for delta check calculation. However, based on a recently published study, no significant change is detected in the CV_i_ values of some analytes (including creatinine) between 18-365 days (*20*). In another publication, it was revealed that delta check rule performance is not affected by the time interval and error detection capacity can be increased by expanding the time period (*21*). Therefore, in our study, when operating delta checks, patients whose previous results were up to one year were included for DPC calculation. As designed in our study, the delta check time interval can be extended up to one year for analytes with low II. Since we used the same method and autoanalyser one year ago, we were able to determine the delta check period as one year to include more patient results in our MPFDC model considering the data in the literature. Patients whose previous results exceeded the one-year period were not included in the model. Since we set the duration to one year, we have reached a quite good number of patients for delta check in our model (67% of patients). As expected, there is some difference between ANDed-ANPed and MNDed-MNPed, but considering the one-year delta check period, we believe this difference appears to be reasonable.

For PBQC applications to be effective, false alarms should not be excessive, and false negatives also should be minimized. In addition, each laboratory should have a scheme for what to do when a PBCQ alarm arises. If the delta check limits or control limits are set low, they may give numerous false alarms (*24*). On the other hand, if the limits are set too high, some errors may not be detected. Therefore, delta check limits and the model’s control limits should be designed well for effective performance. Excessive false alarms may increase workload and turnaround times; it may also be challenging for the staff (*25*). So, we set the bidirectional Z score for RCV calculation as 2.58 to minimize false alarms. If further specificity is desired, optimizations can be attempted by selecting a Z-score of 99.9% instead of 99%, or by requiring the control limit to be reached five times in a row rather than three times to trigger the MPFDC alarm. In our study, for adequate sensitivity, we determined the MPFDC alarm should be triggered by the MPFDC reaching the control limits three times in a row.

When a 15% error was applied in the positive direction, errors were not detected in four batches. These false negatives may have been because of slight negative shifts in these batches that were not detected by internal quality control. We also think the false negatives may have occurred because of the effect of the patient population. Blood may have been predominantly drawn after treatment/hydration in hospitalized patients. When we examined the eight alarms that occurred in 55 batches due to false positive batches, we observed no problems. We suspected the false positives were due to oscillations observed in the data related to the patient population. We carefully reviewed the patient results and the information in the patient files and did not detect any analytical errors in the false alarms.

In our study, for calculating RCV, the CV_i_ value in healthy individuals was used, which may be considered as a limitation. A substantial part of biological variation studies has been conducted with healthy individuals. Some studies have observed that the CV_i_ values of analytes in chronic stable diseases are close to the values of healthy patients (*14*, *26*). However, it has also been shown that CV_i_ values for creatinine may vary in some populations, and disease conditions (*26*-*29*). Our hospital is a tertiary referral center, and there are various subpopulations in our study, such as pediatric, older adult, pregnant, transplantation, intensive care and even nephrology department’s patients. Individuals with the disease, except dialysis patients, were included in our study. Therefore, pathologic changes in creatinine results are commonly seen in our patient population. This may have caused the cutoff we determined for MPFDC to be high. Indeed, using data from biological variation studies that include patient data will make the models more suitable for real settings. Recently, in a striking study, RCV values were created from real patient results by evaluating laboratory data from middleware for nearly two years (*30*). In the study, the RCV value for creatinine obtained from patient data expressed as RCV alternate was found to be 25.3% (*30*). It would be useful for each laboratory to calculate similar data by exhibiting this approach in the studies to be performed in the laboratory.

Laboratories are like factories. Large-scale laboratories may have multiple production lines (*i.e.* instruments) to produce the same product (*i.e.* a measurand). The laboratories are responsible for ensuring the quality of the tests coming out of each analyser in real time and without producing too many problematic results at the time of error detection. If a laboratory applies the MPFDC model by including data from several analysers, an error affecting all instruments can be detected faster as the data stream to the system will increase. However, if just one device from multiple devices produces erroneous results, the erroneous results may be diluted in the data generated from the whole devices, causing the error detection time to extend (*31*). In our study, data flowed from four autoanalysers. Using our error simulation, we simulated a situation that could affect the results across all four devices, namely, an analytical variation, such as deterioration in water quality, inappropriate ambient temperature, improper operating temperatures, use of inappropriate calibrators or internal quality control, problems with reagent preparation, dilution errors, issues with the reagent lot, changes in reagent formulations by the manufacturer, analytical shifts such as drift or shift in calibration, using buffers with the wrong pH, or deterioration in reagents and calibrators during storage or use. The model can also detect preanalytical issues such as inappropriate centrifugation and the detection of incompatible blood samples from external centers (*e.g.*, delayed centrifugation). However, a common situation is that only one of the four devices produces erroneous results, which can lead to dilution of the erroneous results (*31*). This is one of the major limitations of our study. We did not perform the study on an analyser basis. A key point here is that if the MPFDC is operated without distinguishing between all devices in the laboratory, erroneous results from only one device may be diluted, leading to relatively late detection of the error. The number of autoanalysers included in the model may also be important in determining the desired cutoff value for warnings. In the MPFDC model, the cutoffs when the model is run individually for each device may differ from those when all devices in the laboratory are operated together. Similarly, if data flows from multiple devices, increasing the block size for error detection might be a consideration. In fact, we believe that implementing our model on a device-by-device basis would be more beneficial, as it would allow for earlier error detection and could also directly point to the source of the error (the analyser producing the erroneous results). We have not yet tested this in our study, but it could be tested in future studies.

We implemented the MPFDC model in our hospital to the laboratory information system (LIS) (Nucleus, Monad Software and Consultancy, Ankara, Turkey), without the use of a middleware. Information technology staff implemented the model into the LIS within one month. In our hospital, the MPFDC model operates independently of conventional internal quality control as a supplementary tool. A specificity of 86% equals to a false rejection rate of 14% may be considered high for PBQC studies. However, that performance is at level of 15% error. High sensitivity (96%) ensures minimal risk of missing critical problematic events. In our setting, this trade-off provides safer and more clinically relevant performance. Moving percentage of failed delta checks operates continuously in the background, and when an alarm is triggered, a notification is sent to mobile phones of three laboratory specialists. Upon receiving an alert *via* SMS notification, patient results are immediately evaluated by the in-charge laboratory specialist for that day. Data are retrieved *via* LIS in one minute, and we can transfer it to Excel and filter by analyser to see if violations are arising from one analyser. Totally, this process is completed in 5 minutes. After evaluating the patient results on a device-specific basis, and, if deemed necessary, quality control is performed on the relevant device or devices. Also, in future we are planning to do separate analyser monitoring from LIS. Now, we only do separate monitoring by filtering the data, after alarm arises.

The reasons for our ATed being relatively high compared to MTed might be the lower frequency of blood sample arrivals at the laboratory during the night shifts and the skewed distribution of time until error detection. In addition, patients presenting at night and weekends are more probable to be emergent first time-presenting patients and therefore less probable to have previous results and caused higher detection times. In fact, considering that we receive a continuous flow of patient results from four devices, and that approximately 1100 creatinine tests are processed within an 8-hour period, we believe that the ANPed, MNPed, ATed, and MTed values are quite good. The number of samples received *per* hour decreases significantly during the night shift, particularly between 1:00 AM and 6:00 AM. The difference between ATed and MTed stems from the longer error detection times during periods of relatively low sample flow, such as after midnight.

An interesting finding of our study is the differences were observed in the model’s performance when positive and negative errors were applied. This may be due to interventions such as treatment or hydration, patients coming in groups from specific clinics, or shifts too small to be detected by internal quality control, which minimally impact the data. The literature also notes that whether the applied error is positive or negative can affect error detection in various parameters (*5*).

However, there are several limitations of applying PBQC. There is growing interest in PBQC; nevertheless, there are currently still several drawbacks to widespread implementation of PBQC (*32*). Knowledge of statistics and particular abilities of LIS and middlewares are crucial to optimise and implement a PBQC model. Therefore, when designing models, it is important that they are relatively simple, feasible and pragmatic. Another limitation is that our model might have suffered from the “population problem” like any other PBQC techniques (*5*). Variations in the population by time of day, week or month according to specific clinics (nephrology, oncology, pediatrics) due to phlebotomy schedules may not be distinguished from analytical shifts. When applying PBQC, data from particular clinics may be excluded, but this approach may cause a reduction in the data, thus may lag the detection of error. In our study, there were no exclusions based on age or location/ward except dialysis clinic. Thus, we enabled the system to evaluate more patient results. Methods such as truncation limits may reduce the impact of population variation (*5*). Since the number of patients arriving daily is quite high, meaning we are a large-scale hospital, we had the opportunity to perform truncation for those with creatinine results < 27 and > 362 μmol/L. A limitation of this study is the lack of daily inter-instrument comparability assessment. Although identical analytical platforms, reagents, and methods were used, a formal inter-instrument comparison was not performed. Therefore, potential minor instrument-specific biases related to calibration, maintenance conditions, or operator variability could not be excluded. Nevertheless, analytical performance was continuously monitored through internal QC materials run at two-hour intervals for each analyser. In addition, for each instrument, internal quality control means and SD values, instrument CVs, and median values of patient results were presented as supportive indicators of analytical stability.

For measurands, the PBQC models are not one-size-fits-all (*33*). It would be useful to develop unique and distinct PBQC models for each measurand considering factors such as the measurand’s analytical performance, II, and whether the analyte results are normally distributed (*5*, *33*). If delta check is used in a PBQC model, the performance of the model may be affected by the patient population characteristics of the laboratory. In choosing a PBQC model for an analyte, one must first determine how the results for that measurand are distributed. For instance, the MA method performs inadequately for creatinine and ALT, which have right-skewed distributions (*5*). Moreover, for measurands with a low II the delta check implementation is substantial (*14*). Therefore, for these measurands, a PBQC model that includes delta check practices and is based on biological variation may be valuable and should be implemented in laboratories (*32*). Creatinine with a skewed distribution and a low II is suitable for our model. In addition, an advantage of our model is that it does not require a transformation of the patient data. A limitation was that we could not compare the performance of our model with another model, such as MA or MM. Because the creatinine data were already skewed, applying MA without transformation was clearly not feasible.

Particularly for the laboratories performing autoverification, our model may be used as an alarm system for detecting systematic errors in time. If algorithms that enable the detection of irregular analytical errors is already included in the autoverification model, it may be possible to integrate MPFDC into the autoverification plan additionally. In this way, under the guarantee of early awareness of systematic error thanks to our model, this may help broaden the autoverification range, autoverify the patients without previous results and improve autoverification performance. Soon the laboratory users will get more advanced PBQC options. In this context, we call on LIS and middleware vendors to implement PBQC literature and carefully evaluate the documents and spreadsheets shared in literature (*34*). This will allow for maximum security in autoverification applications, thus improving patient safety.

In conclusion, MPFDC a novel RCV-based PBQC model, has high sensitivity and rapid error detection. It evaluates the sustainability of analytical performance quality by considering within-subject biological variation and detecting systematic analytical errors. MPFDC may help as a gatekeeper to support and assist autoverification systems for detecting analytical errors. Particularly, the measurands with a low II are suitable for this model.

## Data Availability

The data generated and analyzed in the presented study are available from the corresponding author on request.
